# Enhanced Respiratory Sound Classification Using Deep Learning and Multi-Channel Auscultation

**DOI:** 10.3390/jcm14155437

**Published:** 2025-08-01

**Authors:** Yeonkyeong Kim, Kyu Bom Kim, Ah Young Leem, Kyuseok Kim, Su Hwan Lee

**Affiliations:** 1 Division of Pulmonology and Critical Care Medicine, Department of Internal Medicine, Yonsei University College of Medicine, 50-1, Yonsei-ro, Seodaemun-gu, Seoul 03722, Republic of Korea; dusrud026@yonsei.ac.kr (Y.K.); yimayoung@yuhs.ac (A.Y.L.); 2 2TS Corporation, 211, Hwarang-ro, Seongbuk-gu, Seoul 02772, Republic of Korea; ssmakal@yonsei.ac.kr; 3 Department of Radiation Convergence Engineering, Yonsei University, 1, Yeonsedae-gil, Heungeopmyeon, Wonju-si 26493, Republic of Korea; 4 Institute of Human Convergence Health Science, Gachon University, 191, Hambakmoe-ro, Yeonsu-gu, Incheon 21936, Republic of Korea

**Keywords:** multi-channel lung sound, deep learning, mel-frequency cepstral coefficient, abnormal respiratory sounds, clinical implication

## Abstract

**Background/Objectives**: Identifying and classifying abnormal lung sounds is essential for diagnosing patients with respiratory disorders. In particular, the simultaneous recording of auscultation signals from multiple clinically relevant positions offers greater diagnostic potential compared to traditional single-channel measurements. This study aims to improve the accuracy of respiratory sound classification by leveraging multichannel signals and capturing positional characteristics from multiple sites in the same patient. **Methods**: We evaluated the performance of respiratory sound classification using multichannel lung sound data with a deep learning model that combines a convolutional neural network (CNN) and long short-term memory (LSTM), based on mel-frequency cepstral coefficients (MFCCs). We analyzed the impact of the number and placement of channels on classification performance. **Results**: The results demonstrated that using four-channel recordings improved accuracy, sensitivity, specificity, precision, and F1-score by approximately 1.11, 1.15, 1.05, 1.08, and 1.13 times, respectively, compared to using three, two, or single-channel recordings. **Conclusions**: This study confirms that multichannel data capture a richer set of features corresponding to various respiratory sound characteristics, leading to significantly improved classification performance. The proposed method holds promise for enhancing sound classification accuracy not only in clinical applications but also in broader domains such as speech and audio processing.

## 1. Introduction

Auscultation is the most basic diagnostic method for respiratory diseases, as it is noninvasive, fast, real-time, and efficient [1,2]. The ability to pre-identify abnormal respiratory sounds by deducing the various pathological conditions of the lungs and bronchi is crucial for patient care [3,4]. Since the first clinically useful and hygienic stethoscope was introduced in 1816 by René Laënnec, Bowles and Sprague developed the Hewlett–Packard Rappaport–Sprague double-tubed stethoscope, considering the bell and diaphragm, which became the common standard stethoscope [3,4,5]. The classic stethoscope has two primary limitations: (1) inherent inter-listener variability depending on the listener’s experience and knowledge; (2) unquantified measurement of lung sounds.

Despite the long-standing use and historical advancement of stethoscopes, the clinical effectiveness of auscultation remains inconsistent, largely owing to variations in practitioners’ auditory interpretation skills and experience levels. In a study of lung auscultation skills among medical students, pulmonologists, and interns in internal and family medicine, the pulmonologists outperformed the other physicians [6]. Previous studies have explored methods to improve physicians’ chest auscultation skills through web-based resources, task trainers, and simulators using recorded stethoscopes and sound-generating devices [7]. However, limitations persist because the effectiveness of the device depends on individual experience and skill variations. Additionally, the medical community does not use a standardized representation or classification of the characteristics of the human respiratory system [8]. These challenges underscore the urgency for quantifiable and objective methods for analyzing lung sounds beyond the subjective auscultatory skills.

Unquantified lung sound measurements represent a primary cause of reduced diagnostic accuracy. Murphy et al. applied a computer-based recording technique to explore the utility of quantifying lung sounds [9]. They indicated that quantitatively capturing lung sounds, which reflect the underlying lung pathophysiology, would be more useful than analog lung auscultation for the diagnosis and monitoring of cardiopulmonary conditions. Electronic stethoscopes with advanced microsensors have also been investigated. Microphone types are the most common in digital stethoscopes and include transistor-based electret condenser [10], piezoelectric [11], fiber-optic [12], and microelectromechanical system (MEMS) microphones [13]. MEMS microphones have comprehensive advantages over other microphones regarding the signal-to-noise ratio, size, high-temperature and vibration tolerance, and wideband frequency response [14,15]. This digital auscultation method acquires quantified respiratory sound data and makes a diagnostic decision independent of the listener’s experience. The measured digital sound signal has been particularly studied for the correlation between each respiratory disease and various features of its signal, including the threshold level for abnormality [16], time frequency and scale analysis [17], skewness and kurtosis [18], and higher-order statistics [19,20].

These features help to reduce the complexity of measured data for disease prediction, making relationships more explicit. Similarly, improved feature extraction methods through domain transformation, such as the short-time Fourier transform and mel-frequency cepstral coefficient (MFCC), are being introduced [21,22]. The accuracy rate of deep learning-based feature extraction and classification for respiratory sounds (normal, crackles, wheezes, and rhonchi) was 85.7%, higher than that for medical students (60.3%), interns (53.4%), residents (68.8%), and fellows (80.1%) [23,24]. However, disturbances in the mechanical properties of the lung parenchyma that are challenging to recognize or a medium with an acoustic impedance different from that of the normal parenchyma between the sound source and the stethoscope hinder the accurate identification of lung sounds using single-channel auscultation [1].

Simultaneous (or multi-channel) recording of clinically useful auscultatory positions has increased the potential for lung diagnostics compared with the effectiveness of traditional single-channel measurements [25]. The characteristics of the lung sound signals recorded at each measurement location differ, facilitating disease prediction. A 16-channel automatic sound analyzer effectively predicted crackle sounds (*r* = 0.74, *p* < 0.001, number of participants = 41) [26]. Another examination using a multi-channel lung sound analyzer has been shown to be feasible, with a sensitivity of 0.84, specificity of 0.94, and positive predictive power of 0.93 in a computer-assisted classification between normal individuals and patients with pneumonia [27,28].

Table 1 summarizes representative studies on respiratory sound classification. While traditional methods such as those of Murphy et al. [28] rely on hardware-based multi-channel systems, recent approaches by Kim et al. [23] and Messner et al. [25] have considered deep learning with varying channel inputs. In addition, Jayalakshmy et al. [29] demonstrated the advantage of synthetic data augmentation in overcoming class imbalance. These studies underscore the growing emphasis on spatial and statistical diversities in classifying respiratory sounds.

Although previous studies have demonstrated the feasibility of using handcrafted statistical features [21] or deep learning with multi-channel inputs [25,26], they face limitations such as restricted scalability, hardware complexity, or insufficient temporal modeling. To address these gaps, we proposed a deep learning-based classification framework that integrates multi-channel auscultation signals and MFCC feature extraction, which was optimized via a hybrid convolutional neural network (CNN)–long short-term memory (LSTM) model. This approach enhances the robustness and clinical applicability of respiratory sound classification systems, particularly in real-world noisy and multi-positional environments. In contrast to Murphy et al. [28], who used single-channel analysis for pneumonia detection, we leveraged simultaneous multi-channel auscultation to capture positional diversity in respiratory sounds. Furthermore, while Jayalakshmy et al. [29] improved classification accuracy using synthetic data generated by conditional generative adversarial networks (cGANs), their reliance on artificial signals may compromise clinical reliability. Our method emphasizes real-world data collection with simple augmentation strategies, enhancing classification performance and translational feasibility in clinical settings.

Compared with the method of existing studies that primarily considers single-channel recordings or synthetic augmentation, our approach incorporates real multi-channel data acquired in a clinical setting. Furthermore, while several models employ CNNs or recurrent neural networks (RNNs) separately, the combined CNN–LSTM architecture used in this study enables spatial and temporal feature learning, which is particularly beneficial for characterizing complex respiratory cycles. These distinctions position our study as a meaningful advancement in practical and robust respiratory sound classification. In this study, we aimed to compare the performance of deep learning-based respiratory sound classification algorithms with multi-channel lung sounds with existing single-channel respiratory sound classification algorithms.

## 2. Materials and Methods

### 2.1. Dataset of Multi-Channel Respiratory Sound

The chest auscultation data used in this study were obtained from a large-scale public dataset funded by the Ministry of Science and ICT of Korea and supported by the National Information Society Agency of Korea, with 25,195 auscultations from 6000 patients (AI-Hub, South Korea, https://www.aihub.or.kr). In contrast to publicly available datasets such as ICBHI 2017 [30], this dataset comprises multi-channel auscultation recordings from real patients. Owing to ethical and privacy considerations, the dataset is not openly downloadable; however, it is accessible upon institutional review at designated research facilities. The ICBHI 2017 [30] dataset comprises data measured for multiple areas, but the total number of patients measured is 126, and the number of patients measured for three or four areas is less. A small number of data can lead to overfitting of the results. The dataset used in this study averages hundreds to thousands of patients who have measured three or four areas. This dataset was designed to overcome the paucity of high-quality, large-scale multi-channel respiratory sound data and improve the accuracy and clinical applicability of AI models.

Each respiratory sound dataset comprises a wav file of approximately <0.5 MB and was recorded by taking >3 deep breaths at least thrice (10 s in total) at >4 of the 12 clinical auscultation positions, including the right upper lung (RUL), left upper lung (LUL), right mid lung (RML), left mid lung (LML), right lower lung (RLL), and left lower lung (LLL) in anterior (front) and posterior (back) views. The measurement equipment used to collect the respiratory sound data were a JABES electronic stethoscope (GS technology, Seoul, South Korea) and Smartsound (Skeeper SM300, Seoul, South Korea). They created a handmade web-based annotation tool based on Label Studio (HumanSignal Inc., San Francisco, CA, USA, https://labelstud.io/) for labeling and divided the respiratory sounds into fixed types, including normal (61.76%), fine crackles (1.96%), coarse crackles (0.13%), rhonchi (30.97%), wheezing (0.04%), and those which could not be analyzed (5.13%). The recordings were collected using a digital stethoscope developed for multi-site auscultation, under the supervision of board-certified respiratory physicians. This ensured that all results presented in this study were not simulated data but were rather derived from actual patient sound signals acquired in a clinical environment. We categorized respiratory sounds into three classes: normal, crackles, and wheezes. This tripartite classification was considered based on their high clinical relevance and prevalence in respiratory diseases such as COPD, pneumonia, and asthma. Mixed or ambiguous sounds were excluded to improve inter-class distinction and annotation reliability. Ultimately, we classified three respiratory sound types, excluding categories with insufficient samples (e.g., coarse crackles and rhonchi).

Following the dual review of the labeling results, labels with annotator disagreements were excluded. The institutional review board of Severance Hospital approved the study protocol (IRB No. 4-2024-1433; 7 January 2025).

**Figure 1:**
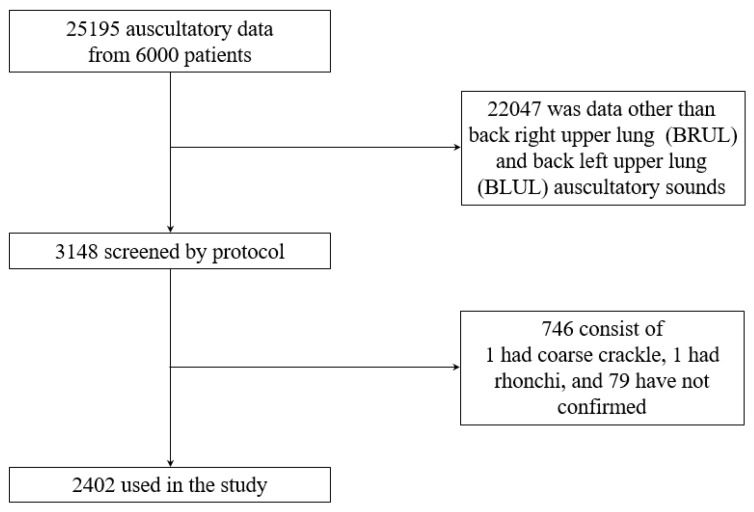
Composition of retrospectively acquired respiratory sound data to predict the multi-channel respiratory sound in the back right upper lung (BRUL) and back left upper lung (BLUL). Here, we obtained 25,195 auscultation data from the first 6000 patients and used a total of 2402 multi-channel auscultations for the study.

The amount of respiratory sound data was analyzed according to the auscultation position to classify the auscultation sounds into multiple channels. Figure 1 shows the process of selecting the multi-channel data of the back-RUL (BRUL) and back-LUL (BLUL) with the most measured data. A total of 3148 auscultatory sounds were measured in the BRLL and BLUL positions, of which 2402 respiratory sounds were used, excluding 1 coarse crackle, 1 rhonchi, and 79 sounds that could not be analyzed. Of the 2402 respiratory sounds, 1827 were normal, 43 were fine crackles, and 447 were wheezing. Over 2000 two-channel respiratory sounds were recorded: BRUL-BLUL, BRUL-back LLL (BLLL), BLUL-BLLL, BRUL-back RLL (BRLL), BLUL-BRLL, and BLLL-BRLL. Three- and four-channel respiratory sounds had the most BRUL-BLUL-BLLL combinations, with 1935 data points, and BRUL-BLUL-BLLL-BRLL combinations with 1660 data points, respectively. Respiratory sounds with >5 channels were excluded because they contained <100 trainable data points.

Data augmentation techniques were used to the time stretching, adding masking noise, pitch shifting, signal conversion, amplitude shifting, adding background noise, Gaussian noise, and time stretching to the raw audio [31]. In addition, recorded ventilation sounds, conversation sounds, and collar rustling sounds that may occur in the ICU, augmented them with conventional methods, and added background noise to the respiratory sound as shown in Equation (1) [32]:

(1) $$I_{out}=\left(1-w\right)\times I_{in}+w\times I_{noise},$$

Here, w is the weight used to balance the measured respiratory sound, $I_{in},$ and the augmented background noise. Here, w ranges between 0 and 1. To address class imbalance in the training data, we augmented underrepresented crackle and wheeze samples by combining time-domain transformations (e.g., noise addition and temporal shifting) and replication. These measures ensured that the model learned meaningful features across the three respiratory sound categories, regardless of their natural distribution in the dataset. These strategies aimed to ensure that the model did not converge toward dominant class predictions and maintained robustness across the three classes. We performed 30 and 4 augmentations on the wheezing and fine crackle data, respectively. Based on the augmented data, we split the data into a 70:15:15 ratio for training, validation, and testing, respectively.

### 2.2. Proposed Multi-Channel Respiratory Sound Classification Based on Deep Learning

**Figure 2:**
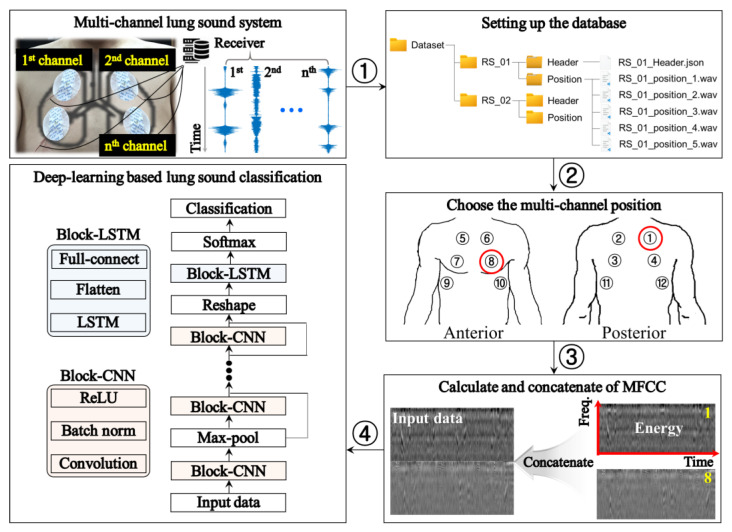
Proposed lung respiratory sound classification scheme implemented using multi-channel respiratory sound with the mel-frequency cepstral coefficient (MFCC).

Figure 2 shows the proposed multi-channel respiratory sound classification scheme.

Briefly, a database was built based on the lung sound signals measured at each location. For each patient, the database organized the header information and raw data of lung sounds by location and stored them (①). The header file contains patient personal information, such as sex, age, weight, underlying disease, and measurement time of multi-channel lung sounds. Raw data is stored as a type of time series in the wav file format. Among the respiratory sounds measured at various positions, it is selected to be used for prediction (②), and the MFCC was calculated in the selected sound data (③). The Mel-frequency cepstrum (MFC) is a linear transformation of the logarithmic energy spectrum based on the nonlinear Mel scale of the sound frequencies. MFCC is the coefficient of MFC. They provide more features than do time-series signals, improving the classification accuracy [33]. In this study, Mel spectrogram-based MFCC features were selected due to their perceptual alignment with human hearing, particularly in the lower frequency bands where adventitious respiratory sounds are prominent. MFCCs offer a more compact and noise-robust representation than do other spectrograms. Here, MFCCs based on multi-channel lung sounds were concatenated in the raw axis direction into a deep learning model. The MFCC features were extracted using a 4 kHz sampling rate, 25 ms window size, 10 ms hop length, and 40 Mel filters. The resulting 13 MFCCs (excluding the 0th) per frame were used as model input.

The model performs classification by combining a CNN based on a residual network (ResNet) [34] and LSTM [35]. ResNet is a network that introduces a residual block to facilitate performance even if the neural networks are deeper. It is defined as indicated in Equation (2):

(2) $$y=F\left(x,\left\{W_{i}\right\}\right)+x,$$

where *x* is the previously learned data, F(·) is a function of deriving the result based on the weight *W* for the *i*-th residual by learning the residual part, and then *y* is derived to add the result of F(·) and existing *x* to the next layer. Residual learning can be trained more effectively than the existing plain learning methods. Here, the network used as a backbone used ResNet-18. This can be changed to suit the needs of the user. LSTM is a type of RNN that addresses the challenge of long-term dependency and comprises cells and gates. The proposed respiratory sound classification model considers LSTM as a useful model to process time-series data and sequence information. To effectively model the spatiotemporal characteristics of respiratory sounds, a hybrid architecture combining CNN and LSTM was adopted. While CNN layers extract localized features from the MFCC spectrograms, the LSTM layers capture temporal dynamics across respiratory cycles. This combination allows the model to recognize spatially distinguishable patterns (e.g., frequency modulations) and time-dependent characteristics (e.g., crackles vs. wheezes), which are critical for accurate respiratory sound classification.

This network contains 57 layers with convolution, batch normalization, ReLU, max, average pooling, LSTM, full connection, and softmax [36]. The overall learning parameter of the model used was 3.6 M. The input layer was set to 128 × 350 pixels, the convolution filter was 7 × 7 and 3 × 3 pixels, the max pooling layer was 3 × 3 pixels, and ReLU-based activation and output layers predicted three classes (i.e., normal, crackle, and wheezing). The LSTM structure was set to one stacked LSTM layer, 64 hidden units per layer, and 0.3 dropout. Furthermore, the final classifier comprised a fully connected layer and a softmax output layer for multi-class prediction. The epoch was set to 50, the batch size was 20, a loss function was used for categorical cross-entropy, and a rectified Adam (RAdam) optimizer (epsilon = 1 × 10^−6^) was used to update the parameters in the back-propagation [37]. The learning rates ranged from 0.0001 to 0.00005. Finally, the classification of respiratory sounds was performed using multi-channel lung sound signals with a pre-trained model (④).

Based on the above descriptions, we implemented measured multi-channel lung sounds and the proposed algorithm. The proposed framework was implemented using a standard workstation (OS: Windows 10, CPU: AMD Ryzen 7 3700X, RAM: 256 GB, GPU: Titan Xp 12 GB), MATLAB software (R2021a, MathWorks Corp., Natick, MA, USA), and PyTorch software (version 2.0.1, Meta AI, Menlo Park, CA, USA).

### 2.3. Evaluation Factors

The confusion matrix, accuracy, precision, recall, and F1-score were used as quantitative evaluation factors. The confusion matrix tool aids in evaluating a classification model using matching between actual and predicted classes, and the accuracy, precision, recall, and F1-score can be defined as indicated in Equations (3)–(6) [38].

(3) $$\mathrm{Accuracy}=\frac{TP+TN}{TP+FP+FN+TN},$$

(4) $$\mathrm{Precision}=\frac{TP}{TP+FP},$$

(5) $$\mathrm{Recall}=\frac{TP}{TP+FN},$$

(6) $$F1\text{-}\mathrm{score}=2\times \frac{Precision\times Recall}{Precision+Recall},$$

where *TP* is a true positive, *FP* is a false positive, *FN* is a false negative, and *TN* is a true negative. The F1-score is defined as a harmonic mean, as an index that considers precision and recall simultaneously, that has a value between 0 and 1; the closer it is to 1, the better the classification performance.

## 3. Results

The classification results for each augmentation method were obtained using the CNN–LSTM model with cross-validation during the initial parameter-tuning steps. The validation accuracies were determined for each configuration across the three randomized training sessions. The evaluation results presented in this study are the ensemble averages of the outcomes of three training sessions. The normal, wheezing, and fine crackle data were labeled as “0”, “1”, and “2”, respectively.

**Figure 3:**
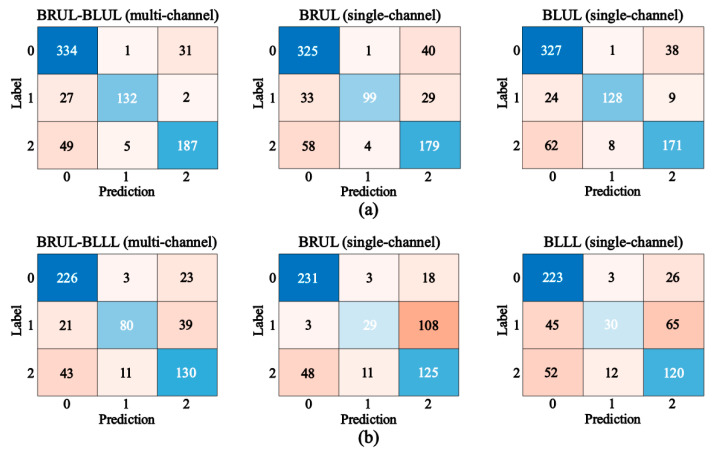
Examples of the confusion matrix to compare the classification accuracy of multi-channel and single-channel in (**a**) BRUL-BLUL positions and (**b**) BRUL-BLLL positions, respectively.

Figure 3 shows the confusion matrix used to compare the classification accuracy of the multi-channels and each single channel in (a) BRUL-BLUL and (b) BRUL-BLLL positions. The accuracy of respiratory sound classification based on the BRUL-BLUL multi-channel was approximately 0.85, higher than those of the single-channel BRUL and BLUL, which were approximately 0.79 and 0.82, respectively. The sensitivity, specificity, precision, and F1-score of the multi-channel lung respiratory sound classification were approximately, 0.84, 0.91, 0.87, and 0.85, respectively, which were approximately 1.08, 1.03, 1.05, and 1.08 times higher than those of the single-channel. The result of the five evaluation factors of the BRUL-BLLL multi-channel data was approximately 1.17 times higher than those of the BRUL and BLLL single-channel data. Table 2 summarizes the accuracy, sensitivity, specificity, precision, and F1-score of the two- and single-channel respiratory sound classifications at the BRUL, BLUL, BLLL, and BRLL positions.

**Figure 4:**
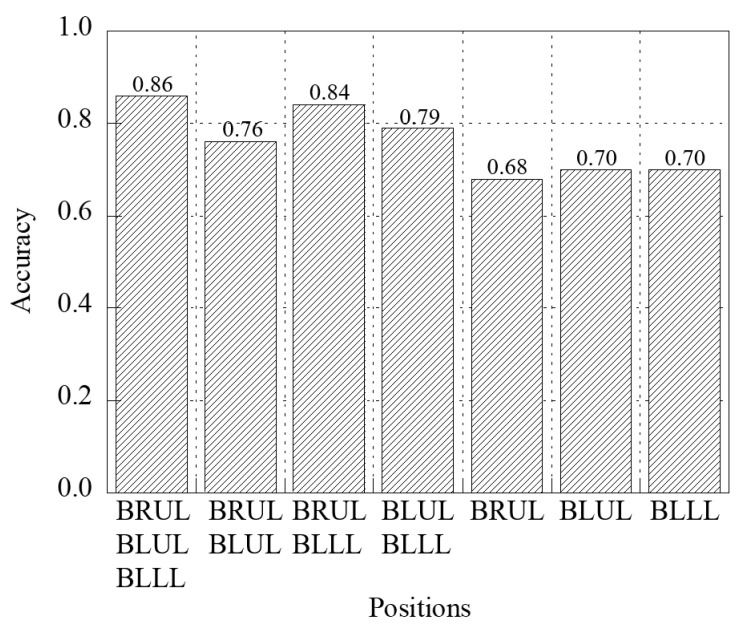
Bar graphs of the accuracy of classifying respiratory sounds in three-channel, two-channel, and single-channel at BRUL, BLUL, and BLLL positions.

Figure 4 shows the accuracy bar graphs for the three-, two-, and single-channel respiratory sounds at the BRUL, BLUL, and BLLL positions. The accuracies of the three- and two-channel respiratory sound classification were approximately 0.86 and 0.80, respectively, which were 1.25 and 1.0 times higher than the average value of approximately 0.69 for the single-channel respiratory sound classification. The sensitivity, specificity, precision, and F1-score were higher for the three-channel respiratory sound classification than for the two- and single-channel models. The prediction results of the respiratory sounds at the BRUL-BLLL positions were the most similar to the prediction results of the three channels. However, this tendency does not necessarily indicate where the BRUL and BLLL auscultation positions improve classification accuracy over other auscultation positions. Table 3 presents the results computed using five evaluation matrices for the three-, two-, and single-channel respiratory sound classifications at the BRUL, BLUL, and BLLL positions, respectively.

**Figure 5:**
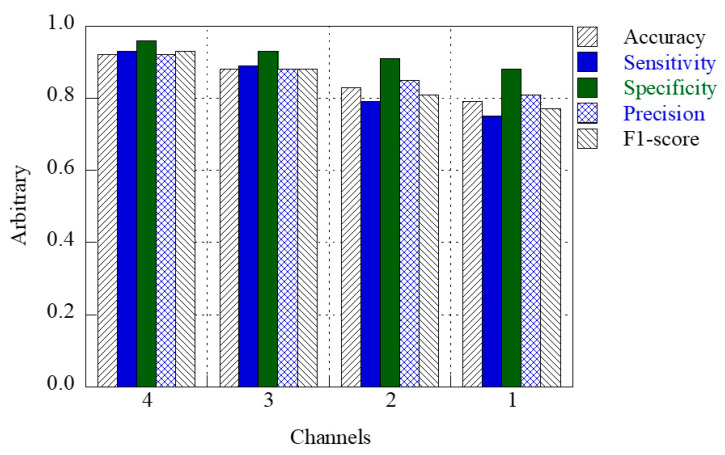
Graphs representing the results of representative four-, three-, two-, and single-channel with the highest values of the five evaluation matrices at the BRUL, BLUL, BLLL, and BRLL positions.

Figure 5 presents a bar graph representing the results of the representative four, three, two, and single channels with the highest values of accuracy, sensitivity, specificity, precision, and F1-score for BRUL, BLUL, BLLL, and BRLL. Here, the selected three, two, and single positions were BRUL-BLLL-BRLL, BRUL-BLUL, and BRLL, respectively. The accuracy of respiratory sound classification was approximately 0.92 for four channels, compared with approximately 0.79, 0.83, and 0.88 for a single, two, and three channels, respectively. Table 4 summarizes the quantitative evaluations of one, two, three, and four channels for representative positions based on the lung sounds measured at BRUL, BLUL, BLLL, and BRLL. The results demonstrate that prediction based on multi-channel respiratory sounds has higher accuracy than that of the single-channel prediction and that respiratory sound classification accuracy improves significantly with an increase in the number of channels.

Table 5 presents the results of respiratory sound classification through time series respiratory-based CNN and CNN–LSTM models and an MFCC-based CNN model to confirm whether the proposed CNN–LSTM with MFCC model benefits respiratory sound classification. For a four-channel time series input value, it was input as 12,000 × 4 pixels and converted into 128 × 350 pixels to adapt it for the CNN model, similar to the existing CNN–LSTM model. The output section was placed at the end of the CNN model and consisted of flatten, fully connected, and output (based on softmax activation that derives 3 units) layers. All models were trained using the Adam optimizer with the learning rates of 0.0001–0.00005, a batch size of 20, and a maximum of 50 epochs. The categorical cross-entropy loss function was used for multi-class classification. To ensure consistency across experiments, we retained identical CNN feature extraction layers and adjusted only the input format (e.g., time-series vs. MFCC) and temporal module (e.g., with or without LSTM).

The CNN model trained on raw time-series inputs achieved an average accuracy of 0.65 ± 0.05, with a limited sensitivity of 0.58 ± 0.07 and F1-score of 0.57 ± 0.05, indicating insufficient detection of positive cases. The CNN–LSTM model using the same time-series input showed improved performance across all metrics, particularly in demonstrating a sensitivity of 0.72 ± 0.04 and an F1-score of 0.73 ± 0.10, suggesting that temporal sequence modeling enhanced discriminative capability. When MFCC features were applied to the CNN model, substantial gains were observed, yielding an accuracy of 0.85 ± 0.06, a sensitivity of 0.84 ± 0.04, and an F1-score of 0.82 ± 0.10. These highlight the effectiveness of MFCC-based feature extraction in representing respiratory characteristics. Notably, the proposed CNN–LSTM model using MFCC input outperformed all configurations. These results demonstrate the synergistic effect of combining MFCC-based acoustic features with temporal modeling via LSTM, validating the effectiveness of our proposed method for respiratory sound classification.

**Figure 6:**
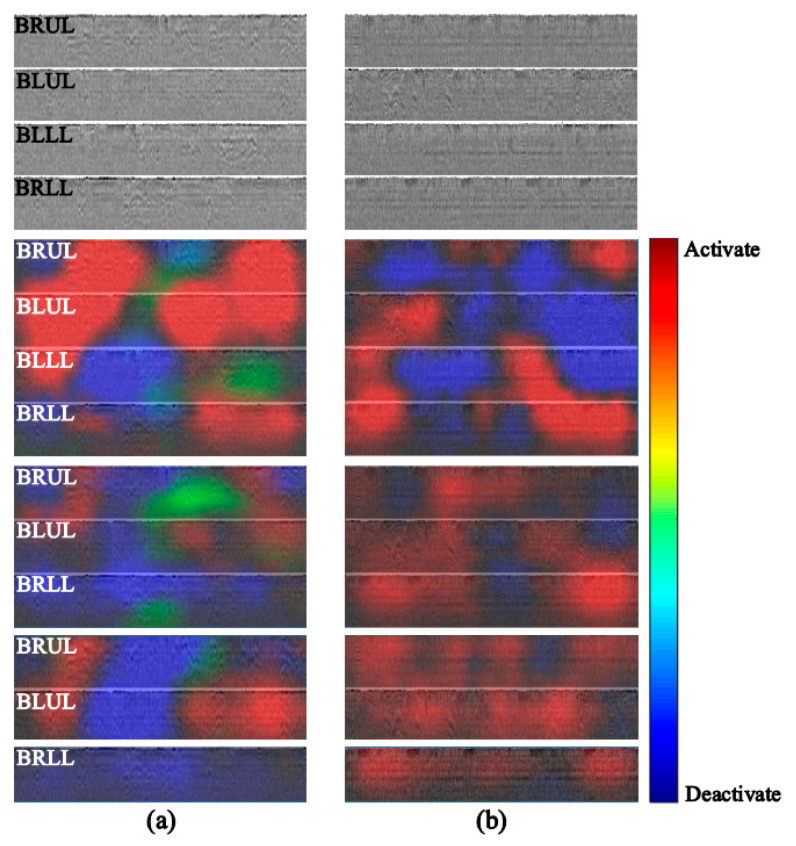
MFCC maps of four-channel lung sound and activation maps of representative four-, three-, two-, and single-channel-based sounds. Here, (**a**) only the four-channel- based lung sounds correctly predicted normal lung sounds and (**b**) only the four-channel- based lung sounds correctly predicted fine crackle. The auscultated positions are BRUL, BLUL, BLLL, and BRLL.

Figure 6 shows the MFCC results and activation maps for four channels that correctly predicted (a) normal sounds and (b) fine crackle sounds, as well as activation maps for one, two, and three channels that made inaccurate predictions. A higher weight (red) indicates that the model referenced that region more accurately than the others when classifying respiratory sounds [39]. For the activation map of the four-channel lung sound that predicted correctly, a certain proportion of regions were referenced to derive the results. However, as the number of channels decreased, most of these tended to be referenced without distinguishing features. Moreover, the feature space used for the accurate prediction was unclear in activation maps with a single channel or a few channels. Nevertheless, the feature space for the correct prediction became finer-grained as the number of channels increased. While the region used to predict accurately was unclear in the activation maps of a single or two channels, those of three or four channels showed that the feature region was refined by considering the auscultation signals from different positions. The deep learning model is expected to predict results accurately and reliably when based on multi-channel lung sounds.

## 4. Discussion

The proposed deep learning-based prediction study using multi-channel lung sounds showed the following primary results:

(1) The predictive classification rate and accuracy improved as the number of channels used to measure respiratory sounds increased. Multi-channel lung sounds contained more features for each respiratory sound, allowing the classifier to perform accurate classifications. This facilitates an accurate classification in cases with numerous external noise signals.
(2) The differences in sensitivity and specificity decreased with multi-channel respiratory sound classification. This indicates that the prediction method is more reliable than single-channel respiratory sound classification and that multi-channel auscultation minimizes the loss of information and acquires more characteristic data on respiratory sounds than single-channel auscultation. This minimizes the dependence of prediction on the auscultation position and is meaningful as standardized respiratory sound classification data.
(3) The F1-score was higher for multi-channel lung sound-based predictions than for other approaches, and each respiratory sound classification was independent of the position. This indicates that the multi-channel respiratory sound classification has higher accuracy and reproducibility, independent of specific locations. The feasibility of the multi-channel lung sound-based prediction method for predicting respiratory diseases in clinical practice was confirmed.

Therefore, the proposed respiratory sound classification method proved to be more practically useful than single-channel respiratory sound classification in the quantitative and qualitative aspects.

However, there are certain limitations. Firstly, multi-channel lung sounds were lacking, and each lung sound had an unbalanced ratio. The lung sound database had different sizes (1.5–1500), depending on the class. The most authoritative large public database (ICBHI 2017 Challenge) contains 6898 respiratory cycles and 920 single-channel recordings [30]. These are insufficient to predict respiratory disease and constrain the dataset to train multi-channel lung respiratory sound classification models, as the data were obtained from measuring only a few positions. If the results are compared with previous studies based on the published dataset, the reliability of the proposed method can be improved, but the lack of datasets that can compare and verify significant levels of multi-channel respiratory sound measurement-based respiratory sound prediction accuracy is a major limitation. To overcome this challenge, data augmentation focuses on a possible approach, including classical (i.e., time stretching, pitch shifting, and dynamic range compression) and machine learning-based methods [40]. The attention mechanism-based respiratory sound classification contributes to improving the average score by 2.95% and 0.84% on the validation and official test sets, respectively [41]. The automatic analysis of lung diseases with domain transform data using the ICBHI 2017 Challenge dataset achieved a sensitivity of 54% and a specificity of 42% [42]. Jayalakshmy et al. showed a classification accuracy of 92.50% using the Resnet-50 model with cGAN-based augmentation, compared with the 81.37% accuracy without augmentation, 80.17% with sequential categorical-based augmentation, and 85.40% with independent categorical-based augmentation [29]. These encouraging results indicate that data augmentation techniques are useful for improving the classification accuracy of simultaneous lung sound signals.

Second, a major limitation of this study lies in the restricted number of respiratory sound types used for classification. The dataset was annotated with only three categories: normal, crackles, and wheezes. While these are among the most common auscultatory findings, they do not encompass the entire clinical spectrum of adventitious lung sounds. Other significant sounds such as rhonchi, stridor, pleural rubs, and fine versus coarse crackles were not included owing to limited labeled data and inter-observer dissensus. This reduction in class diversity may cause overfitting to the available classes and misclassification of atypical or mixed sounds in real-world cases [43]. Additionally, some patients may exhibit overlapping characteristics between classes, further complicating accurate labeling and recognition. A more granular or hierarchical classification scheme might better reflect the nuanced nature of respiratory acoustics. However, this would require a larger and more carefully annotated dataset. Future work should expand the class structure to improve the clinical utility and diagnostic specificity of the model.

Finally, significant external noise from the intensive care unit (ICU) environment should be eliminated. The noise level in the ICU is notably high owing to the presence of various monitoring devices and many clinical staff, potentially distracting them from monitoring the patients on ventilators. Excessive sound pressure levels in ICUs frequently fall within the range of 50–70 dB (A), and levels exceeding 40 dB (A) impede concentration [44,45]. Deep learning-based methods have been introduced as noise reduction methods and exhibit high removal performance [46]. It exhibits a high noise-cancelation effect within a given condition, primarily by canceling noise based on previously obtained data. However, the reproducibility of results cannot be guaranteed for untrained data. A dual microphone-based noise cancelation method may overcome this challenge [47,48]. By employing an adaptive blocking matrix and adaptive noise cancelation to reduce noise in real time, they demonstrated the possibility of improving the voice quality of the measured breathing sounds. We plan to continue research and development to overcome these limitations.

## 5. Conclusions

We classified respiratory sound using multi-channel lung sound signals with an MFCC. The purpose was to improve the classification accuracy of lung sounds using multi-channel signals to optimally capture the characteristics specific to each measurement position on the same patient. We investigated the number and location of effective channels. The results indicated that four-channel lung sound prediction improved accuracy, sensitivity, specificity, precision, and F1-score by approximately 1.11, 1.15, 1.05, 1.08, and 1.13 times, respectively, compared with the three-, two-, and single-channel results. The difference between sensitivity and specificity decreased for the multi-channel respiratory sound-based prediction, indicating that the method is more reliable. Furthermore, the proposed multi-channel lung sound-based prediction had a high F1-score, which is expected to improve the accuracy and reproducibility of future respiratory sound classifications. The proposed research method is a result of measurements using several respiratory sound measurement devices, and the developed real-time multi-channel respiratory sound measurement system will be considered in clinical trials. Additionally, it is expected to contribute to improving the diagnostic accuracy because it is compatible with various modalities, such as electrocardiography.

## Figures and Tables

**Table 1:** Representative previous studies related to respiratory sound classification methods, highlighting their methodologies, strengths, and limitations. This provides the basis for the proposed multi-channel approach in this study.

Study	Method	Strengths	Limitations
[28]	Multi-channel lung sound analyzer with computerized acoustic processing	Enhanced detection of adventitious sounds (e.g., crackles) with quantitative analysis	Requires specialized multi-channel stethoscope hardware and controlled environment
[23]	Deep learning classification using single-channel auscultation data (CNN-based)	Automated detection of crackles, wheezes, and rhonchi in clinical settings	Lacks spatial context and robustness to positional variation
[25]	Convolutional Recurrent Neural Network (CRNN) using multi-channel data	Captures both spatial and temporal features with high classification accuracy	High computational cost and need for large-scale annotated data
[29]	Conditional GAN-based data augmentation with ResNet-50 for classification	Overcomes class imbalance and enhances model generalization	Complex training pipeline and requires GAN tuning expertise

**Table 2:** Performance comparison of two-channel and single-channel respiratory sound classification among the positions of BRUL, BLUL, BLLL, and BRLL.

Positions	Accuracy	Sensitivity	Specificity	Precision	F1-Score
BRUL-BLUL (2-ch.)	0.85 ± 0.03	0.84 ± 0.02	0.91 ± 0.07	0.87 ± 0.05	0.85 ± 0.09
BRUL (single)	0.79 ± 0.01	0.75 ± 0.09	0.88 ± 0.03	0.82 ± 0.02	0.77 ± 0.06
BLUL (single)	0.82 ± 0.05	0.80 ± 0.12	0.89 ± 0.05	0.84 ± 0.07	0.81 ± 0.06
BRUL-BLLL (2-ch.)	0.75 ± 0.08	0.72 ± 0.11	0.87 ± 0.03	0.77 ± 0.03	0.74 ± 0.05
BRUL (single)	0.67 ± 0.06	0.60 ± 0.03	0.83 ± 0.12	0.66 ± 0.02	0.59 ± 0.10
BLLL (single)	0.65 ± 0.05	0.58 ± 0.13	0.81 ± 0.13	0.64 ± 0.06	0.57 ± 0.03
BLUL-BLLL (2-ch.)	0.76 ± 0.02	0.72 ± 0.10	0.87 ± 0.07	0.76 ± 0.03	0.73 ± 0.05
BLUL (single)	0.63 ± 0.06	0.71 ± 0.09	0.80 ± 0.05	0.71 ± 0.04	0.68 ± 0.11
BLLL (single)	0.70 ± 0.05	0.68 ± 0.12	0.85 ± 0.04	0.73 ± 0.01	0.70 ± 0.05
BRUL-BRLL (2-ch.)	0.80 ± 0.01	0.81 ± 0.05	0.88 ± 0.03	0.87 ± 0.09	0.82 ± 0.08
BRUL (single)	0.68 ± 0.02	0.77 ± 0.03	0.75 ± 0.03	0.75 ± 0.10	0.77 ± 0.05
BRLL (single)	0.76 ± 0.06	0.75 ± 0.12	0.73 ± 0.02	0.75 ± 0.02	0.75 ± 0.06
BLUL-BRLL (2-ch.)	0.73 ± 0.01	0.70 ± 0.08	0.75 ± 0.03	0.73 ± 0.06	0.71 ± 0.03
BLUL (single)	0.68 ± 0.04	0.65 ± 0.02	0.70 ± 0.03	0.70 ± 0.05	0.65 ± 0.01
BRLL (single)	0.68 ± 0.02	0.67 ± 0.05	0.60 ± 0.02	0.65 ± 0.05	0.60 ± 0.01
BLLL-BRLL (2-ch.)	0.71 ± 0.02	0.73 ± 0.05	0.78 ± 0.03	0.71 ± 0.05	0.73 ± 0.08
BLLL (single)	0.68 ± 0.01	0.75 ± 0.07	0.81 ± 0.02	0.60 ± 0.03	0.70 ± 0.04
BRLL (single)	0.70 ± 0.04	0.76 ± 0.07	0.75 ± 0.05	0.68 ± 0.05	0.70 ± 0.07

**Table 3:** Performance evaluation of three-, two-, and single-channel respiratory sound prediction among the positions of BRUL, BLUL, and BLLL.

Positions	Accuracy	Sensitivity	Specificity	Precision	F1-Score
BRUL-BLUL-BLLL (3-ch.)	0.86 ± 0.09	0.87 ± 0.01	0.93 ± 0.01	0.87 ± 0.02	0.87 ± 0.02
BRUL-BLUL (2-ch.)	0.76 ± 0.03	0.73 ± 0.06	0.88 ± 0.04	0.76 ± 0.02	0.74 ± 0.03
BRUL-BLUL (2-ch.)	0.84 ± 0.02	0.84 ± 0.05	0.92 ± 0.02	0.83 ± 0.01	0.83 ± 0.03
BRUL-BLUL (2-ch.)	0.79 ± 0.08	0.78 ± 0.02	0.89 ± 0.01	0.78 ± 0.03	0.78 ± 0.04
BRUL (single)	0.68 ± 0.08	0.61 ± 0.11	0.83 ± 0.05	0.83 ± 0.04	0.59 ± 0.02
BLUL (single)	0.70 ± 0.03	0.64 ± 0.06	0.84 ± 0.06	0.73 ± 0.06	0.63 ± 0.02
BLLL (single)	0.70 ± 0.07	0.65 ± 0.06	0.85 ± 0.05	0.72 ± 0.01	0.66 ± 0.08

**Table 4:** Evaluate the prediction performance of four-channel, three-channel, two-channel, and single-channel lung sounds representative of the BRUL, BLUL, BLLL, and BRLL positions.

Positions	Accuracy	Sensitivity	Specificity	Precision	F1-Score
BRUL-BLUL-BLLL-BRLL (4-ch.)	0.92 ± 0.02	0.93 ± 0.02	0.96 ± 0.05	0.92 ± 0.01	0.93 ± 0.03
BRUL-BLLL-BRLL (3-ch.)	0.88 ± 0.04	0.89 ± 0.02	0.93 ± 0.06	0.88 ± 0.05	0.88 ± 0.02
BRUL-BLUL (2-ch.)	0.83 ± 0.01	0.79 ± 0.06	0.91 ± 0.07	0.85 ± 0.03	0.81 ± 0.09
BRLL (single)	0.79 ± 0.02	0.75 ± 0.10	0.88 ± 0.03	0.81 ± 0.02	0.77 ± 0.04

**Table 5:** Comparison results of a convolutional neural network (CNN), CNN–long short-term memory (LSTM), CNN with mel-frequency cepstral coefficient (MFCC), and CNN–LSTM with MFCC models using the four-channel respiratory sounds.

Model	Accuracy	Sensitivity	Specificity	Precision	F1-Score
CNN	0.65 ± 0.05	0.58 ± 0.07	0.81 ± 0.03	0.64 ± 0.03	0.57 ± 0.05
CNN–LSTM	0.76 ± 0.01	0.72 ± 0.04	0.87 ± 0.06	0.76 ± 0.02	0.73 ± 0.10
CNN with MFCC	0.85 ± 0.06	0.84 ± 0.04	0.88 ± 0.11	0.85 ± 0.04	0.82 ± 0.10
CNN–LSTM with MFCC	0.92 ± 0.02	0.93 ± 0.02	0.96 ± 0.05	0.92 ± 0.01	0.93 ± 0.03

## Data Availability

The raw data supporting the conclusions of this article will be made available by the authors on request.

## References

[1] Bohadana A., Izbicki G., Kraman S.S. (2014). Fundamentals of lung auscultation. N. Engl. J. Med..

[2] Sarkar M., Madabhavi I., Niranjan N., Dogra M. (2015). Auscultation of the respiratory system. Ann. Thorac. Med..

[3] Bishop P.J. (1980). Evolution of the stethoscope. J. R. Sco. Med..

[4] Roguin A. (2006). Rene theophile hyacinthe laënnec (1781–1826): The man behind the stethoscope. Clin. Med. Res..

[5] Cushman W.C., Cooper K.M., Horne R.A., Meydrech E.F. (1990). Effect of back support and stethoscope head on seated blood pressure determinations. Am. J. Hypertens..

[6] Mangione S., Nieman L.Z. (1999). Pulmonary auscultatory skills during training in internal medicine and family practice. Am. J. Respir. Crit. Care. Med..

[7] Ward J.J., Wattier B.A. (2011). Technology for enhancing chest auscultation in clinical simulation. Respir. Care.

[8] Hafke-Dys H., Bręborowicz A., Kleka P., Kociński J., Biniakowski A. (2019). The accuracy of lung auscultation in the practice of physicians and medical students. PLoS ONE.

[9] Murphy R.L.H., Sorensen K. (1973). Chest auscultation in the diagnosis of pulmonary asbestosis. J. Occup. Med..

[10] Li S.H., Lin B.S., Tsai C.H., Yang C.T., Lin B.S. (2017). Design of wearable breathing sound monitoring system for real-time wheeze detection. Sensors.

[11] Spyropoulos B., Tzavaras A., Afentoulidis P., Botsivaly M. Supporting medical house-call by expanding bedside in-vitro point of care test-range and attaining respiratory sounds’ visualization. Proceedings of the 2013 IEEE Point-of-Care Healthcare Technologies (PHT).

[12] Hayber S.E., Tabaru T.E., Keser S., Saracoglu O.G. (2018). A simple, high sensitive fiber optice microphone based on cellulose triacetate diaphragm. J. Lightwave Technol..

[13] Kusainov R.K., Makukha V.K. Evaluation of the applicability of MEMS microphone for auscultation. Proceedings of the 2015 16th International Conference of Young Specialists on Micro/Nanotechnologies and Electron Devices.

[14] Bogue R., Du H. (2007). MEMS sensors: Past, present and future. Sens. Rev..

[15] Lee S.H., Kim Y.S., Yeo W.H. (2021). Advances in microsensors and wearable bioelectronics for digital stethoscopes in health monitoring and disease diagnosis. Adv. Healthc. Mater..

[16] Sakai T., Kato M., Miyahara S., Kiyasu S. Robust detection of adventitious lung sounds in electronic auscultation signals. Proceedings of the 21st International Conference on Pattern Recognition (ICPR2012).

[17] Serbes G., Sakar C.O., Kahya Y.P., Aydin N. (2013). Pulmonary crackle detection using time-frequency and time-scale analysis. Digit. Signal Process..

[18] Ash S.Y., Harmouche R., Vallejo D.L.L., Villalba J.A., Ostridge K., Gunville R., Come C.E., Onieva J.O., Ross J.C., Hunninghake G.M. (2017). Densitometric and local histogram based analysis of computed tomography images in patients with idiopathic pulmonary fibrosis. Respir. Res..

[19] Naves R., Barbosa B.H.G., Ferreira D.D. (2016). Classification of lung sounds using higher-order statistics: A divide-and-conquer approach. Comput. Methods Programs Biomed..

[20] Pramono R.X.A., Bowyer S., Rodriguez-Villegas E. (2017). Automatic adventitious respiratory sound analysis: A systematic review. PLoS ONE.

[21] Sengupta N., Sahidullah M., Saha G. (2016). Lung sound classification using cepstral-based statistical features. Comput. Biol. Med..

[22] Jung S.Y., Liao C.H., Wu Y.S., Yuan S.M., Sun C.T. (2021). Efficiently classifying lung sounds through depthwise separable CNN models with fused STFT and MFCC features. Diagnostics.

[23] Kim Y., Hyon Y., Lee S., Woo S.D., Ha T., Chung C. (2022). The coming era of a new auscultation system for analyzing respiratory sounds. BMC Pulm. Med..

[24] Kim Y., Hyon Y., Jung S.S., Lee S., Yoo G., Chung C., Ha T. (2011). Respiratory sound classification for crackles, wheezes, and rhonchi in the clinical field using deep learning. Sci. Rep..

[25] Messner E., Fediuk M., Swatek P., Scheidl S., Smelle-Juttner F.M., Olschewski H., Pernkopf F. (2020). Multi-channel lung sound classification with convolutional recurrent neural networks. Comput. Biol. Med..

[26] Murphy R.L., Del Bono E.A., Davidson F. (1989). Validation of an automatic crackle (rale) counter. Am. Rev. Respir. Dis..

[27] Murphy R.L.H., Vyshedskiy A., Power-Charnitsky V.A., Bana D.S., Marinelli P.M., Wong-Tse A., Paciej R. (2004). Automated lung sound analysis in patients with pneumonia. Respir. Care.

[28] Murphy R. (2007). Computerized multichannel lung sound analysis. Development of acoustic instruments for diagnosis and management of medical conditions. IEEE Eng. Med. Biol. Mag..

[29] Jayalakshmy S., Sudha G.F. (2021). Conditional GAN based augmentation for predictive modeling of respiratory signals. Comput. Biol. Med..

[30] Rocha B.M., Filos D., Mendes L., Vogiatzis I., Perantoni E., Kaimakamis E., Natsiavas P., Oliveira A., Jacome C., Marques A. (2018). A respiratory sound database for the development of automated classification. ICBHI 2017: Precision Medicine Powered by pHealth and Connected Health.

[31] Li X., Zhang W., Ding Q., Sun J.Q. (2020). Intelligent rotating machinery fault diagnosis based on deep learning using data augmentation. J. Intell. Manuf..

[32] Abeysinghe A., Tohmuang S., Davy J.L., Fard M. (2023). Data augmentation on convolutional neural networks to classify mechanical noise. Appl. Acoust..

[33] Chu H.C., Zhang Y.L., Chiang H.C. (2023). A CNN sound classification mechanism using data augmentation. Sensors.

[34] He K., Zhang X., Ren S., Sun J. (2015). Deep residual learning for image recognition. arXiv.

[35] Hochreiter S., Schmidhuber J. (1997). Long short-term memory. Neural Comput..

[36] Shrestha A., Mahmood A. (2019). Review of deep learning algorithms and architectures. IEEE Access.

[37] Liu L., Jiang H., He P., Chen W., Liu X., Gao J., Han J. (2021). On the variance of the adaptive learning rate and beyond. arXiv.

[38] Paul C., Bora P. (2021). Detecting hate speech using deep learning techniques. Int. J. Adv. Comput. Sci. Appl. (IJACSA).

[39] Selvaraju R.R., Cogswell M., Das A., Vedantam R., Parikh D., Batra D. Grad-CAM: Visual explanations from deep networks via gradient-based localization. Proceedings of the 2017 IEEE International Conference on Computer Vision (ICCV).

[40] Salamon J., Bello J.P. (2017). Deep convolutional neural networks and data augmentation for environmental sound classification. IEEE Signal Process. Lett..

[41] Yang Z., Liu S., Song M., Parada-Cabaleiro E., Schuller B.W. (2020). Adventitious respiratory classification using attentive residual neural networks. Proc. Interspeech.

[42] Minami K., Lu H., Kim H., Mabu S., Hirano Y., Kido S. Automatic classification of large-scale respiratory sound dataset based on convolutional neural network. Proceedings of the 2019 19th International Conference on Control, Automation and Systems (ICCAS).

[43] Rocha B.M., Filos D., Mendes L., Serbes G., Ulukaya S., Kahya Y.P., Jakovljevic N., Turukalo T.L., Vogiatzis I.M., Perantoni E. (2019). An open access databased for the evaluation of respiratory sounc classification algorithms. Physiol. Meas..

[44] Tainter C.R., Levine A.R., Quraishi S.A., Butterly A.D., Stahl D.L., Eikermann M., Kaafarani H.M., Lee J. (2016). Noise levels in surgical ICUs are consistently above recommended standards. Crit. Care Med..

[45] Vreman J., van Loon L.M., van den Biggelaar W., van der Hoeven J.G., Lemson J., van den Boogaard M. (2020). Contribution of alarm noise to average sound pressure levels in the ICU: An observational cross-sectional study. Intensive Crit. Care Nurs..

[46] Dubey H., Gopal V., Cutler R., Aazami A., Matusevych S., Braun S., Eskimez S.E., Thakker M., Yoshioka Y., Gamper H. Icassp 2022 deep noise suppression challenge. Proceedings of the ICASSP 2022—2022 IEEE International Conference on Acoustics, Speech and Signal Processing (ICASSP).

[47] Zhou Y., Wang H., Chu Y., Liu H. (2021). A robust dual-microphone generalized sidelobe canceller using a bone-conduction sensor for speech enhancement. Sensors.

[48] Yang C., Dai N., Wang Z., Cai S., Wang J., Hu N. (2023). Cardiopulmonary auscultation enhancement with a two-stage noise cancellation approach. Biomed. Signal Process. Control.

